# Measuring disease activity and predicting response to intravenous immunoglobulin in chronic inflammatory demyelinating polyneuropathy

**DOI:** 10.1186/s40364-019-0154-2

**Published:** 2019-02-12

**Authors:** Anthony Khoo, Joseph Frasca, David Schultz

**Affiliations:** 1 Department of Neurology, Flinders Medical Centre, Bedford Park, South Australia 5042 Australia; 2 College of Medicine and Public Health, Flinders University, Adelaide, South Australia

**Keywords:** Chronic inflammatory demyelinating polyneuropathy, Intravenous immunoglobulin, Biomarkers, Paranodal antibodies, Nerve ultrasound, Magnetic resonance neurography

## Abstract

Chronic inflammatory demyelinating polyneuropathy (CIDP) is characterised by significant clinical heterogeneity and as such reliable biomarkers are required to measure disease activity and assess treatment response. Recent advances in our understanding of disease pathogenesis and the discovery of novel serum-based, electrophysiologic and imaging biomarkers allow clinicians to make more informed decisions regarding individualised treatment regimes.

As a chronic immune-mediated process typified by relapse following withdrawal of immunomodulatory therapy, a substantial proportion of patients with CIDP require long term treatment with intravenous immunoglobulin (IVIg), a scarce and expensive donor-derived resource. The required duration and intensity of immunoglobulin treatment vary widely between individuals, highlighting both the heterogeneous nature of the underlying disease process as well as the variable pharmacologic properties of IVIg.

This review outlines the use of multimodal biomarkers in the longitudinal evaluation of nerve injury and how recent developments have impacted our ability to predict both response to immunoglobulin administration and its withdrawal.

## Background

Chronic inflammatory demyelinating polyneuropathy (CIDP) is the most common chronic immune-mediated neuropathy worldwide, with an estimated prevalence of 2–7 per 100,000 people [1]. While therapeutic advances have resulted in a reduction in morbidity, CIDP continues to be associated with considerable disability, with almost three-quarters of patients not returning to their previous level of function [2]. Although effective, traditional corticosteroid regimes are implicated in a wide variety of adverse effects and consequently a substantial proportion of patients require long term treatment with intravenous immunoglobulin (IVIg), a scarce donor-derived resource both costly and inconvenient [3].

The current approach to diagnosis and treatment of CIDP has several significant limitations. While foremost amongst these is the lack of a safe, efficacious and readily accessible therapy, the lack of effective disease biomarkers has resulted in an inability to stratify patients to individualised treatment regimes. While clinical response to treatment can provide an indication of disease activity, objective measures of assessing therapeutic effect and predicting outcome are limited, and this has many implications on assessing the required duration and intensity of immunoglobulin therapy.

### Diagnosis and clinical staging of CIDP

The initial diagnosis of CIDP is clinical, with patients presenting with a characteristic pattern of weakness and areflexia that evolves over a period of more than 2 months. Confirmation of diagnosis is made by demonstrating evidence of peripheral nerve demyelination, most commonly by electrophysiological testing with supportive findings on cerebrospinal fluid analysis and rarely nerve biopsy.

In lieu of well-defined objective measures of disease activity, a variety of scoring systems based on functional status have been developed to quantify disease severity. Commonly used scales include the Inflammatory Neuropathy Cause and Treatment (INCAT) overall disability sum score which relies on self-reported impairment in undertaking activities of daily living and the Medical Research Council (MRC) Muscle Sum Score which relies on examiner-based evaluation of strength in a variety of muscles (See Table 1) [4–8].

**Table 1:** Clinical scoring systems in Chronic Inflammatory Demyelinating Polyneuropathy

Clinical scoring system	First published	Range	Examines	Scored by
MRC Muscle Sum Score	Kleyweg et al., 1991 [4]	0–60	Strength (0–5) in six muscles (both sides): - Deltoid - Biceps - Wrist extensors - Iliopsoas - Quadriceps - Tibialis anterior	Examiner
INCAT Overall Disability Sum score	Merkies et al., 2002 [5]	0–12	Upper and lower limb functional questionnaire	Patient
Adjusted INCAT	Identical to INCAT disability score but changes in upper limb function from 0 (normal) to 1 (minor symptoms) excluded.			
INCAT sensory subscore	Merkies et al., 2000 [8]	0–20	Sensory modalities in upper and lower limb areas and 2-point discrimination at index finger	Examiner
Overall Neuropathy Limitations Scale	Graham et al., 2006 [7]	0–12	Modified ODSS which also includes question regarding running or climbing stairs	Patient
Rasch-built Overall Disability Scale	Van Nes et al., 2011 [6]	0–24	Upper and lower limb functional questionnaire; score of 0–2 in 12 activities eg. washing, climbing stairs	Patient

Abbreviations: *MRC* Medical Research Council, *INCAT* Inflammatory Neuropathy Cause and Treatment, *ONLS* Overall Neuropathy Limitations Scale

While reflecting functional status is important, clinical scores are imperfect approximations of disease activity and their utility in guiding therapeutic decisions in a complex heterogeneous condition like CIDP is clearly limited. Despite consensus guidelines, rates of misdiagnosis in CIDP may exceed 40%, and an over-reliance on self-reported treatment benefits may lead to inappropriate utilisation of resources and subjecting patients to unnecessary treatment risks [9].

Reliable biomarkers of disease activity are thus required to not only aid diagnosis, but also monitor longitudinal disease activity and predict individual responses to both immunoglobulin treatment or its withdrawal.

### Biomarkers of disease activity

A heterogeneous disease process that affects patients to different degrees of severity, various pathogenic mechanisms have been suspected to drive peripheral nerve demyelination in CIDP. While the presence of inflammatory infiltrates on sural nerve biopsies implicate a cell-mediated immune response, early animal-based studies demonstrating that inoculation of sera from CIDP patients produced a demyelinating phenotype and the proven efficacy of plasma exchange in treatment strongly suggest that humoral autoimmunity underpins disease pathogenesis [10, 11].

#### Serum based biomarkers of disease activity

Extensive attempts to identify antibodies against myelin based protein peptides have been largely unrewarding [12]. Studies examining other neuronal structures however, with particular scrutiny on proteins associated with the nodal and paranodal junctions have yielded more promising results. Indeed, while pathogenesis in CIDP has traditionally been conceptualised as being purely myelin based, it is becoming increasingly evident that ‘demyelination’ may be a more complex phenomenon that also involves a disruption of nodal and paranodal regions [13].

The discovery of paranodal antibodies to neurofascin and contactin-1 isoforms have been described in a minority of patients with severe CIDP and the presence of these antibodies appear to predict a phenotype characterised by aggressive symptom onset, sensory ataxia and poor response to IVIg [14, 15]. Identification of these antibodies has provided the first direct evidence of disease-specific biomarkers that provide a tantalising step forwards into the realm of individualised treatment regimes.

Anti-neurofascin 155 (anti-NF155) and anti-contactin 1 (anti-CNTN1) antibodies have been identified in approximately 3–10% of patients with chronic infammatory polyneuropathies [16–18]. Patients who tested positive to these paranodal antibodies responded favourably to B-cell depleting therapies like rituximab over more traditional therapeutic options like IVIg or plasmapheresis. Although only small groups have been studied, a correlation between antibody titre and disease activity has been observed, with successful treatment characterised by a concomitant reduction in antibody levels suggesting these titres could also be used to monitor progress over time [19].

Testing for different immunoglobulin classes of paranodal antibodies may be useful in evaluating patients with a phenotype of aggressive, younger-onset inflammatory neuropathy (even if this resembles a Guillain-Barré Syndrome) particularly in the setting of either treatment resistance or clinical relapse following an initial response to IVIg therapy. While transient IgM responses to neurofascin can be seen in patients with GBS, the presence of IgG4 antibodies appears to be extremely specific for an eventual diagnosis of CIDP [17, 18]. It could be hypothesized that presence of paranodal antibodies of the IgM class may increase risk of progression to CIDP (IgM class switching is mandatory for IgG4 antibody formation) and this could be an indication for heightened vigilance even if initial presentation is atypical.

Despite the promise shown by these discoveries, the identification of IgG4 paranodal antibodies in patients with CIDP remains rare, and while early indications of a specificity approaching 100% make them an invaluable tool for assessing patients with suggestive clinical presentations, more ubiquitous biomarkers are clearly necessary for routine clinical use [17].

#### Serologic responses to therapy

Although the scarcity of detectable antibodies in CIDP mean they are an impractical method of measuring disease activity, the quantifiable serologic response to immunoglobulin treatment has been proposed as an alternative surrogate biomarker.

The mechanisms by which IVIg exerts a regulatory effect on the dysimmune response in CIDP has not been completely established, though in-vivo studies have suggested it may neutralize pathogenic autoantibodies, inhibit complement binding and possibly act directly on the myelin sheath to enhance remyelination [20]. These pharmacodynamic mechanisms are driven to different degrees in affected patients, hence explaining individual responses to treatment. More importantly, pharmacokinetic effects directly influencing serum immunoglobulin levels after IVIg administration likely explain the variability in individual responsiveness and the clinical observation that patients experience a ‘wearing-off’ effect at different time-points after treatment [21, 22].

There is mounting evidence to support an immunoglobulin dose-response relationship in inflammatory neuropathies, with higher serum levels after IVIg administration associated with a superior treatment response [23, 24]. Certainly in acute inflammatory demyelinating polyneuropathy, statistically significant associations between change in IgG levels after IVIg and various functional endpoints such as muscle strength and being able to walk unaided have been demonstrated. While the association between change in immunoglobulin G level (∆IgG) and clinical outcome has not been as comprehensively evaluated in CIDP, small group experimental data appears to support a similar positive relationship [25].

Following the results of the Intravenous Immunoglobulin CIDP Efficacy (ICE) trial which established IVIg as an effective therapy for CIDP, the standard approach to initiating treatment has been to commence with an induction dose of 2 g/kg IVIg over 2–5 days. This recommendation however does not take into account patient-specific pharmacokinetic responses, and adjusting treatment dose based on immunoglobulin levels may help guide more effective individualised treatment regimes. A study of change in immunoglobulin G level (∆IgG) following treatment actually demonstated wide variability between patients unrelated to weight or body mass index [21]. As such, although current practice bases total treatment dose on weight, many other factors influence IVIg pharmacokinetics and it is likely that other variables including fraction of dimeric IgG and fragment crystallizable (Fc) receptor polymorphisms have a direct influence on IVIg efficacy and metabolism [26].

Comprising pooled IgG derived from between 1000 and 15,000 different donors, IVIG preparations consist of predominantly monomeric IgG with small percentages (5–15%) of dimeric complexes. This is relevant as increased dimeric fractions have been reported to be associated with increased incidence of adverse effects such as hypotension [27]. Intriguingly however, emerging data among patients with CIDP has also demonstrated significant correlations between dimeric IgG level post-treatment and clinical improvement [28].

Although their clinical utility in routine practice is yet to be established, other emerging immunoglobulin-specific biomarkers include level of sialylated IgG and the ratio of sialylated/agalactosylated IgG-Fc levels. These relate to the percentage of carbohydrate (galactose or sialic acid) binding to the immunoglobulin Fc region, the primary site involved in both complement activation and antibody-dependent cell-mediated cytotoxicity [29]. Patients with CIDP have been shown to have lower serum levels of baseline sialylated IgG and sialylated/agalactosylated IgG-Fc ratios. Furthermore, these biomarkers appear to correlate with pre-treatment disease severity and increase following administration of IVIg [30].

Many individuals with CIDP require long term maintenance treatment with IVIg to effect disease stability and studies of serum IgG levels in treatment responders have revealed that patients actually achieve a steady state wherein both pre- and post-treatment IgG levels are almost identical [23]. The implications of this are profound, as it suggests there is an individual threshold above which patients remain stable [16]. Although at the present time identifying this threshold in any given patient is a matter of trial and error, further validation of reference ranges in which disease control is achieved provides promise for a future wherein IVIg is administered with the aim to achieve pre-specified IgG levels [23].

#### Electrophysiologic biomarkers

While electrophysiology has an established role in the diagnosis of CIDP, its ability to monitor response has not been as clearly defined. Furthermore, the degree of nerve demyelination and axonal injury at diagnosis does not appear to predict either response to treatment or long-term disability [31]. From a longitudinal perspective, while serial nerve conduction studies are objective and reproducible measures of electrophysiologic function, they appear to correlate surprisingly poorly with clinical progression or resolution [32].

Studies involving patients with biopsy proven CIDP demonstrated that while electrophysiologic changes were present in all participants at diagnosis, serial nerve conduction studies at 4, 8 and 12 months did not correlate well with improvement as measured by functional scores [33]. Despite being stable on treatment or even experiencing clinical improvement, most patients did not consistently show normalisation in nerve conduction studies at 12 months, and a significant proportion actually demonstrated deterioration in motor and sensory amplitudes likely reflecting secondary axonal loss.

The discordance between clinical status and neurophysiology was most apparent in motor and sensory studies of median and ulnar nerves in the upper limb [33]. While motor studies in the lower limb did seem to parallel functional status better, this correlation was again unexpectedly poor and sensory studies when obtainable were rarely reflective of interval clinical direction.

This discrepancy can in part be attributed to the poor relationship between sensory deficits and functional impairment. While distal sensory deficits in patients with CIDP often persist and occasionally worsen despite treatment (reflecting a degree of irreversible axonal loss), this rarely contributes to functional disability and as such it comes as no great surprise that the correlation between sensory studies and functional scores is poor [34].

Despite being a chronic disease, long term electrophysiologic monitoring data in CIDP has not been widely published. Observational studies up to 24 months after diagnosis suggest that certain electrophysiologic parameters and in particular resolution of conduction block may be useful in monitoring disease activity but the clinical utility of using such selective electrophysiologic biomarkers is difficult to assess and unlikely to reflect an efficient use of resources [35].

Use of a composite electrophysiologic score assessing averaged compound motor action potential (CMAP) amplitudes in a number of motor nerves appeared to correlate with treatment response in *post-hoc* analyses of the ICE study [36]. Further validation of this and other composite measures such as averaged motor conduction velocities are however required to support the use of this innovative electrophysiologic biomarker.

The approach to patients who remain clinically stable but who develop interval deterioration in nerve conduction studies is controversial. While observational data has suggested that extensive axonal degeneration is a marker of poor outcome, the role for escalating immunotherapy in the setting of clinical stability has not been adequately studied [2]. Although it has been proposed that patients who develop new demyelinating lesions while on maintenance therapy may benefit from higher doses of IVIg to improve function and possibly prevent relapse this runs the risk of significantly over-treating patients who have remained clinically well [37].

Multiple explanations for the disparity between electrophysiologic demyelination and disease activity have been postulated to explain why evidence of new demyelinating lesions does not always parallel clinical decline. CIDP remains a dynamic process that involves constant segmental demyelination and remyelination, and it is likely this constant spectrum of nerve injury cannot be easily assessed through electrophysiology at any single time point. Biopsy studies revealing so-called ‘onion bulb’ formations which represent repetitive Schwann cell induced proliferation provides strong evidence for a continuous regeneration routine at the microscopic level which cannot be easily quantified with the use of nerve conduction studies.

#### Imaging biomarkers

While ‘onion bulb’ formation is a pathologic biopsy finding, there is evidence that quantifiable nerve root hypertrophy as measured by magnetic resonance imaging may be a possible radiologic correlate and the advent of advanced imaging techniques such as nerve ultrasound and magnetic resonance neurography have opened the way for multimodal assessments of nerve pathology [38].

Ultrasonography provides precise measures of nerve anatomy in addition to information regarding fascicular related muscles and blood vessels. Use in compressive neuropathies like carpal tunnel syndrome has highlighted its high sensitivity in detecting subclinical nerve damage even in patients without symptoms [39]. Furthermore, the ability to identify demyelinating neuropathies like Charcot-Marie-Tooth disease and CIDP has been particularly promising, with one study reporting sensitivities of up to 100 and 86% respectively [40].

Specificity in CIDP diagnosis can be increased through the use of ultrasound in addition to electrophysiology. One study comparing patients with diabetic polyneuropathy fulfilling electrophysiologic criteria for CIDP to those with clinically diagnosed CIDP demonstrated that cross-sectional area as measured on ultrasound reliably distinguished these two groups apart [41].

In addition to being a useful adjunct in diagnosis, longitudinal changes in nerve ultrasound characteristics appear to correlate well with clinical status and amelioration of nerve enlargement during treatment with IVIg concordant with clinical improvement may thus be a promising surrogate biomarker of disease stability [42].

There are however several technical factors that can limit ultrasound use. In particular, meaningful sonographic evaluation of the nervous system is only possible at accessible sites in the distal extremities and its ability to accurately visualise proximal structures like the lumbosacral plexus and spinal nerves is extremely limited. This is particularly pertinent in conditions like CIDP, as electrophysiologic markers at disease onset (delayed F-waves and absent H-responses) strongly suggest the process begins as a proximal polyradiculopathy that only subsequently extends distally.

Using magnetic resonance imaging, it is possible to assess these proximal areas and thus identify structural evidence of peripheral nerve pathology. Early MRI studies involving gadolinium enhanced T1-weighted sequences described enhancement of the cauda equina (in up to 70% of cases) and less commonly nerve root enlargement however concluded that these features could not correlate with either clinical signs or disease severity [43]. Modern sequences for MR-neurography however, which include a combination of fat-suppressed T2- and T1-weighted sequences can reliably quantify cross-sectional nerve area and demonstrate nerve signal change, thus providing objective measures of disease activity that can be monitored over time.

As assessed by MR neurography, patients with CIDP had significantly enlarged cross-sectional areas and signal intensity in nerves of the lumbosacral plexus, the sciatic nerve at the level of the thigh and major nerves of the upper limb when compared with normal controls and this suggests it can be used as a highly specific diagnostic aid [44]. While correlations of cross-sectional area and T2 signal intensity with clinical severity failed to reach significance, imaging parameters correlated strongly with neurophysiology markers of nerve injury, in particular lower limb CMAP amplitude and F-wave latency. Consequently, although multiparametric MRI imaging can be used as a highly specific diagnostic tool, its role in estimating severity is yet to be fully elucidated [45].

In addition to examining nerve pathology, adjunctive myopathic imaging in patients with CIDP may help assess disease severity, with structural alterations in muscle tissue composition as a consequence of denervation also correlating closely with clinical weakness [46]. Several studies have demonstrated that thigh muscles of patients with CIDP showed a significantly elevated intramuscular fat fraction compared with controls [45, 46].

While both nerve signal change and structural nerve or muscle measurements provide valuable diagnostic data and indirect estimations of disease activity the emerging use of advanced functional MRI techniques involving diffusion tensor imaging (DTI) promises to yield additional quantitative information which can be used to monitor disease activity in CIDP. By using diffusion sensitizing-gradients to measure proton diffusion within individual voxels DTI allows visualization of nerve fibre tracks and recent studies have shown promise in its ability to provide quantitative data on myelin sheath integrity [45–47].

### Genomics in CIDP: A novel biomarker?

Clinical genomics is starting to play an increasingly important role in our understanding of disease pathogenesis and has offered the potential for an increased application of high-precision medicine in a variety of autoimmune conditions.

Differential gene expression in CIDP as measured through micro-array based analysis of human sural nerve biopsies has provided information on a number of up-regulated genes in these patients [48]. As sural nerve biopsies are rarely performed due to their invasive nature and the potential for permanent complications, subsequent investigations were carried out to see if these upregulated genes could also be identified in skin biopsies.

One study suggested that five genes were reliably upregulated in skin biopsy samples in CIDP patients, with the Allograft Inflammatory Factor-1 gene most significantly associated. While it remains unclear what triggers this upregulation, all these genes have been implicated in the immune cascade [49]. Unfortunately, while significant differences in gene expression have been identified in CIDP patients compared to normal controls, these genes also appear to be upregulated in other conditions like vasculitis and even diabetic neuropathy and thus more specific genetic biomarkers in CIDP are required.

There are early indications that genetic profiling may open novel monitoring strategies during treatment with IVIg. Pilot data involving patients treated with immunoglobulin demonstrated that IVIg drove the down-regulation of a number of genes involved in the systemic inflammatory response however these findings need to be validated in larger populations until more definitive conclusions can be drawn [50].

### Predicting response to IVIG withdrawal

Current criteria for immunoglobulin use mandate considering cessation of IVIg in all patients after 12 months of treatment. Discontinuing immunoglobulin therapy can however be associated with recrudescence of a significant symptom burden, and relapse rates of up to 45% at 6 months have been reported [51]. Of particular concern, the re-introduction of IVIg as rescue therapy in relapsed patients may not always lead to full recovery although the reasons for this are not well elucidated [52]. Consequently, just as reliable biomarkers are necessary to monitor disease activity on IVIg, so too is it crucial to develop the ability to predict response to immunoglobulin withdrawal.

Observational data of clinical characteristics in patients with CIDP revealed no significant difference in topography of weakness (proximal vs. distal), type of neuropathic disturbance (sensory vs motor) or pre-treatment severity between individuals who could be weaned from therapy and those who were treatment dependent [32]. This strongly suggests that using clinical phenotype alone is a poor predictor of response to treatment cessation.

While serum-based and imaging biomarkers have shown promise in being able to quantify disease activity, their role in predicting response to a discontinuation of therapy has not been determined (see Table 2). Conversely, despite some discordance between neurophysiology and predicting clinical outcome, certain electrophysiologic parameters have been shown to be useful in identifying patients at high risk of an inflammatory relapse following withdrawal of treatment.

**Table 2:** Emerging biomarkers in CIDP

Investigation		Comments
Serum biomarkers		
Paranodal antibodies	anti-NF155 IgM	Can be seen in GBS, CMT and CIDP
	anti-NF155 IgG4	Highly specific for CIDP, often characterised by younger age of disease onset, sensory ataxia and poor response to IVIg
	anti-NF186 IgG4	
	anti-NF140 IgG4	
	anti-CNTN1 IgG4	
Serologic response to IVIg	Change in total IgG level	
	IgG dimer index	
	Level of sialylated IgG	
Electrophysiology		
Resolution of conduction block		Correlate well with longitudinal disease activity unlike most other nerve conduction studies
Averaged CMAP amplitude		
Accumulation of demyelinating features		Predictor of relapse following treatment withdrawal
Imaging		
High resolution ultrasound	Cross sectional nerve area	
	Nerve echogenicity	Role still unclear
	Nerve vascularization	
Magnetic resonance neurography	Cross sectional nerve area	
	Nerve signal change	
Functional MRI (Diffusion tensor imaging)	Fractional anisotropy	Correlate well with electrophysiological markers of demyelination
	Radial diffusivity	Role still unclear
	Axial diffusivity	

Abbreviations: *NF* Neurofascin, *GBS* Guillain Barre Syndrome, *CIDP* Chronic inflammatory demyelinating polyneuropathy, *IVIg* intravenous immunoglobulin, *CMAP* compound motor action potential, MRI: magnetic resonance imaging

Standard markers of demyelination on nerve conduction studies include slowing of nerve conduction velocity, temporal dispersion, prolongation of distal motor and F-wave latencies in addition to conduction block. Due to the fact that CIDP is a dynamic disease process that involves perpetual demyelination and remyelination these electrophysiological biomarkers do not necessarily correlate well with functional based clinical scores at individual time points. Nonetheless, use of these markers in stratifying risk of relapse can help influence decision-making by predicting patients who may be good candidates for weaning therapy.

An ICE extension study which randomised treatment responders into either continued IVIg or placebo (i.e. IVIg discontinued) performed a post-hoc analysis of demyelinating changes in patients who subsequently relapsed with electrophysiologic data obtained at the initial commencement of IVIg and just prior to discontinuing treatment [53].

Although limited by small sample size the results obtained in each group at these two time-points were highly suggestive. All patients who relapsed demonstrated evidence of new demyelinating features between the two studies thus indicating that the accumulation of an electrophysiologic burden of disease may predict treatment dependency. Interestingly, 60% of patients in the No-Relapse group also accumulated demyelinating features, which suggests that number of new lesions in itself is not a particularly specific finding. Comparing the actual type of interval demyelinating change between the two groups however suggested that F-wave latency and distal compound motor action potential duration, which had odds ratios of 14.40 and 21.00 respectively, may be more potent biomarkers for predicting relapse following treatment withdrawal [53].

## Conclusion

The search for reliable biomarkers of disease activity in CIDP continues. While the discovery of specific paranodal antibodies which conform to phenotypic presentations of disease and predict response to immunomodulation represents a major advance in our approach to individualised treatment strategies, more ubiquitous biomarkers that provide robust longitudinal measures of neuropathic injury are clearly required.

Immunoglobulin treatment dependence has significant physical and psychological consequences on patients in addition to profound financial implications at a population level, due to both inherent costs of treatment administration but also downstream effects driven by long-term patient disability.

Promising genomic studies, serum-based assays, electrophysiological techniques and imaging modalities are emerging at a rate commensurate with our greater understanding of CIDP pathogenesis. How effectively the use of these various complementary investigations translates into a practical multimodal approach that has the ability to predict an individual’s response to immunoglobulin treatment however still remains to be seen.

## Abbreviations

∆IgG: Change in immunoglobulin G
CIDP: Chronic inflammatory demyelinating polyneuropathy
CNTN1: Contactin-1
DTI: Diffusion tensor imaging
Fc receptor: Fragment crystallizable receptor
GBS: Guillain Barré Syndrome
ICE: Intravenous immunoglobulin CIDP efficacy trial
IgG: Immunoglobulin G
INCAT: Inflammatory Neuropathy Cause and Treatment
IVIg: Intravenous immunoglobulin
MRC: Medical Research Council
MRI: Magnetic resonance imaging
MR-neurography: Magnetic resonance neurography
NF155: Neurofascin-155
ONLS: Overall neuropathy limitations scale
RODS: Rasch-built overall disability scale
